# Formulation of Gluten-Free Cookies Utilizing Chickpea, Carob, and Hazelnut Flours through Mixture Design

**DOI:** 10.3390/foods12193689

**Published:** 2023-10-08

**Authors:** Ilgin Dogruer, Filiz Baser, Sukru Gulec, Figen Tokatli, Banu Ozen

**Affiliations:** 1 Department of Food Engineering, Izmir Institute of Technology, Urla, TR35430 Izmir, Türkiye; ilgindogruer@iyte.edu.tr (I.D.); filizbaser@iyte.edu.tr (F.B.); sukrugulec@iyte.edu.tr (S.G.); figentokatli@iyte.edu.tr (F.T.); 2 Molecular Nutrition & Human Physiology Laboratory, Izmir Institute of Technology, Urla, TR35430 Izmir, Türkiye

**Keywords:** gluten-free, cookie, chickpea flour, carob flour, hazelnut flour, mixture design

## Abstract

Legume flours, which offer high nutritional quality, present viable options for gluten-free bakery products. However, they may have an objectionable flavor and taste for some consumers. In this study, it was aimed to improve the gluten-free cookie formulation by incorporating carob and hazelnut flours to pre-cooked chickpea flour and to investigate the techno-functional properties of the formulated cookies. The flours used in the formulations were assessed for their chemical and physical properties. This study employed a mixture design (simplex-centroid) to obtain the proportions of the flours to be used in the cookie formulations. The rheological characteristics of the doughs and the technological attributes of the baked cookies were determined. The addition of the hazelnut and carob flours had the overall effect of reducing the rheological characteristics of the cookie doughs. Furthermore, the textural attribute of the hardness of the baked cookies decreased as the ratio of hazelnut flour in the formulations was raised. The analysed results and sensory evaluation pointed to a formulation consisting of 30% pre-cooked chickpea/30% carob/30% hazelnut flours, which exhibited improved taste and overall acceptability scores. A total of 16.82 g/100 g of rapidly digestible starch, 5.36 g/100 g of slowly digestible starch, and 8.30 g/100 g of resistant starch exist in this particular cookie. As a result, combinations of chickpea, hazelnut, and carob flours hold promise as good alternatives for gluten-free cookie ingredients and warrant further exploration in the development of similar products.

## 1. Introduction

Lately, there has been a significant rise in the consumption of products that are gluten-free and a market growth of about 10.8% is estimated in gluten-free bakery products between 2022 and 2030 [1]. It has been indicated that the growing popularity of gluten-free diets can be attributed to the improved diagnosis and heightened awareness of gluten-related allergies, intolerance, and sensitivities, or a prevailing belief that gluten-free items offer enhanced health benefits [2,3]. Wheat allergy, non-celiac gluten sensitivity, and celiac disease are the three primary gluten-related conditions that affect a considerable portion of the population [4]. Consuming gluten-containing foods adversely affects the small intestine and diminishes nutritional absorption in individuals with celiac disease. The only remedy for celiac disease and other gluten-related ailments involves eliminating gluten from one’s diet. For people with celiac disease, it is crucial to broaden the variety of gluten-free products. While the market for these items has been constantly growing, the current offerings often lack sufficient nutritional value or fall short in terms of taste when compared to gluten-containing alternatives. Generally, these gluten-free products predominantly include rice flour and starch rather than nutrient-rich legume flours, leading to nutritional deficiencies in the products, particularly in terms of the protein content. Addressing this concern can involve incorporating legume flours and other palatable raw materials into formulations.

Leguminosae, a plant family encompassing approximately 17,600 species and around 690 genera, comprises dicotyledonous seeds known as legumes. These legumes serve as good nutritional sources with a multitude of health benefits. They offer substantial quantities of vitamins, minerals, and complex carbohydrates, alongside proteins, dietary fibers, and various nutrients [5]. In developing nations, legumes hold the position of the second-largest human food source, following cereals, particularly among those with limited incomes. They play a role in diversifying diets and providing cost-effective protein sources for these countries [6].

Chickpea, ranked as the fifth most significant global crop, has remarkable attributes. With notable protein digestibility, substantial complex carbohydrates (yielding a low glycemic index), and a rich vitamin and mineral content, chickpeas exhibit a high nutritional profile [7]. Despite these nutritional features, the distinct taste and aroma of chickpea-based bakery items might contribute to consumer reluctance, owing to unfamiliarity. This study aims to improve the inherent taste and flavor of chickpea flour by incorporating varying proportions of carob and hazelnut flours into cookies. The addition of hazelnut and carob flours holds the potential to enhance consumer acceptance of cookies, simultaneously increasing the functional properties of the product.

Carob flour receives attention due to its notable phenolic content and fiber-rich nature along with its low fat content [8]. Further, its cocoa-like taste and aroma render it an appealing choice for consumers, serving as a successful cocoa alternative in baked goods. With its distinctive chemical composition including proteins, carbohydrates, fats, and vitamins, the hazelnut emerges as a pivotal nut variety [9]. The product known as hazelnut flour is produced by properly crushing natural or roasted hazelnuts and it is one of the most significant of the hazelnut products. The introduction of hazelnut flour to bakery formulations, with its high fat content, elevates the taste, smell, and texture of the end products.

Substituting gluten in food products presents a challenge due to a variety of issues, encompassing nutritional deficiencies and textural characteristics. For instance, formulating doughs with flours not containing gluten is more complex due to their low level of cohesion, flexibility, and optimal baking properties, as highlighted in the literature [10]. Notably, gluten-free products often have high starch and low fiber contents, limited shelf-life, or textural concerns such as denser crumbs [11]. To formulate gluten-free goods with similar characteristics to their wheat-based counterparts, diverse combinations of gluten-free flours and their supplementary components have been investigated [12]. The nutritional advantages of using legume flour must be balanced against the fact that the inclusion of these components reduces the organoleptic attributes of the final product [13]. The combined use of other flours can help mask the off-odors of chickpea flour and minimize the challenges with the acceptability of the baked goods made with it. In previous studies, chickpea flour was used in combination with wheat or chestnut flours and it was stated that the use of composite flours improved the acceptability of the cookies along with the changes in the nutritional and technological properties [14,15].

While the nutritional advantages of using legume flours are evident, the inclusion of these ingredients can sometimes compromise the sensory attributes of the final product. Therefore, the primary aim of this study is to develop cookie formulations with enhanced functional properties by incorporating legume (carob) and nut (hazelnut) flours into pre-cooked chickpea flour through a mixture design approach. Additionally, we aim to comprehensively investigate the rheological, technological, sensory, and digestibility characteristics of these newly formulated cookies. By achieving these objectives, our research seeks to provide nutritious, more palatable, and overall improved formulations from the consumer’s perspective.

## 2. Materials and Methods

### 2.1. Contents of the Formulations

Pre-cooked chickpea and carob flours were obtained from Naturelka (Aydın, Türkiye). Hazelnut flour, produced from raw hazelnuts with their skin intact, and corn starch were procured from Ingro (Karaman, Türkiye). The remaining constituents used in the cookie formulations were obtained from the following local suppliers: eggs (approximately 60 g) (Ercanlar, Izmir, Türkiye); margarine (Sana, Istanbul, Türkiye); brown sugar (Takita, Izmir, Türkiye); and baking powder (Dr. Oetker, Izmir, Türkiye).

### 2.2. Measurement of Flour Characteristics

#### 2.2.1. Chemical Composition

AOAC 925.05, AOAC 960.39, AOAC 950.48, and AOAC 923.03 were used in the assessment of the moisture, fat, protein, and ash levels, respectively. Determination of fiber content was conducted in accordance with AOAC 14.020. Quantification of total phenolic contents was achieved with a spectrophotometric technique detailed in the literature, and findings were quantified in milligrams of gallic acid equivalent/liter [16]. The reported findings represent the averages of three analyses.

#### 2.2.2. Physical Characteristics

Color parameters were determined with a colorimeter (CR-400, Konica Minolta, Tokyo, Japan). A method from the literature was used to measure bulk density [17]. The assessment of water retention capacity was conducted in accordance with AACC Method 56-11. The evaluation of oil absorption capacity, emulsification properties, as well as foam properties, followed established protocols outlined in the literature [18].

### 2.3. Product Formulations

The cookie recipes in this study were derived from a prior study that only used various types of chickpea flour [19]. Cookies having 3 different flours were formulated by using a simplex-centroid mixture design (Table 1). For all formulations, amounts of corn starch, egg, sugar, margarine, and baking powder were the same while the ratio of the flours were changed. Total amount of the flours in all formulations was constant and was 90 g. A mixer (KitchenAid, Benton Harbor, MI, USA) was utilized to thoroughly combine all the ingredients, after which 20 g of dough was molded using a circular cutter (diameter: 5 cm). Subsequently, the doughs were subjected to baking in a convection oven (Std, Senox, Türkiye) (175 °C, 10 min). This process was repeated to obtain two separate batches, each consisting of 10 cookies per batch, for every specific type.

### 2.4. Measurement of Rheological Characteristics

Rheological analysis was performed employing a texture measurement equipment (TA-XT2i, Stable Microsystems, Godalming, UK), using back extrusion technique. Details of the analysis and the measurement parameters were given in the literature [19]. As a result of the measurements, rheological parameters of the doughs as indicated in the literature were determined [20]. Two separate measurements were conducted for each batch of cookies.

### 2.5. Determination of Cookie Characteristics

#### 2.5.1. Quality Characteristics

The moisture levels in the products were measured by subjecting the sample to drying at 105 °C [21]. The degree of baking weight loss (BWL) was quantified by taking the difference of the weights of baked cookie and dough. A caliper was employed to measure dimensions of the cookies, and subsequently, the averages of 10 cookies were taken. The spread factor was defined as diameter/height [14]. A colorimeter was employed in the color measurement of the surface of the product.

#### 2.5.2. Measurement of Texture Characteristics of Cookies

Hardness measurements were carried out on the formulations stored within glass jars, 24 h subsequent to baking. Hardness as a textural attribute was assessed with texture measurement equipment (TA-XT2i, Stable Microsystems, Godalming, UK). A bending test with 3 points was used in the texture analysis. The same test parameters given in the literature were used in the measurements [19]. The peak force value was taken as the hardness parameter at the point of product fracture [22]. The recorded hardness values were the averages from five distinct cookies within each batch.

#### 2.5.3. In Vitro Digestion Test of Cookies

The samples were crushed in a ceramic mortar. Then, the starch fractions in the cookie structure were evaluated using a method outlined in a previously published work [23]. The reported values represent the means of the 3 analyses.

#### 2.5.4. Sensory Evaluation

A sensory test was conducted involving 40 participants with ages ranging from 21 to 56 who were not specifically trained for sensory evaluation. Ethical approval for the sensory test was granted by the Izmir Institute of Technology Scientific Research and Publication Ethics Committee (Approval Number: 19.09.2022-E.96273). Panelists were asked to sign an informed consent form prior to testing. Participants employed a hedonic scale ranging from 1 to 7 to assess three cookie variations based on characteristics like texture, flavor, color, taste, and acceptability. In this sensory test, a score of 1 meant the lowest level of preference, while a score of 7 indicated the highest.

The test was performed in a sensory assessment laboratory. The testing environment had individual panel booths illuminated with white light. To ensure objectivity, each cookie sample was assigned a unique identifier and presented on white plastic plates. The panelists were provided with water between the samples.

### 2.6. Statistical Analysis

The outcomes from the flour analysis, the rheological measurements, technological properties, and sensory evaluation underwent assessment through analysis of variance. Results were evaluated using Minitab software (v. 19, Minitab, Coventry, UK). Subsequently, Tukey analysis was employed for the identification of variations among the distinct types across various attributes, with statistical significance considered for values of *p* < 0.05.

A simplex-centroid mixture design was applied to three independent numeric factors, which were chickpea flour (X1: 15–60% flour + starch basis), carob flour (X2: 15–60% flour + starch basis) and hazelnut flour (X3: 15–60% flour + starch basis) (Table 1). The lower (−1) and upper (1) levels were chosen according to the results of the preliminary cookie making trials. There are 7 levels in the design and one of these levels is the central point with 3 replicates (formulations 7, 8 and 9). The suitability of the model was evaluated by considering the R^2^, adjusted-R^2^, *p*-value, and lack of fit of the model. Insignificant components were removed from the model to obtain a better fit, and the resulting reduced models were used to calculate the presented responses for the best formulations. Due to its capability to offer useful information from a limited number of tests and to analyze the interactions among variables, the mixture design methodology is frequently used to solve the optimization challenges in the food industry [24]. Minitab 19 software (Minitab Inc., Coventry, UK) was used for the construction of the mixture design and statistical assessment of the data.

## 3. Results and Discussion

### 3.1. Characteristics of the Flours

The chemical characteristics of the investigated flours used in the cookie formulations were assessed and the findings can be found in Table 2. Among all the flours used in this study, pre-cooked chickpea flour has the highest protein content. The protein content of chickpea flour was reported as 23.7% in a study in the literature [25] and our results are close to the value given in this study. Hazelnut and carob flours have lower protein contents compared to chickpea flour. The amount of protein in carob flour which was found as 3.66 g/100 g is slightly lower than the value reported as 4.62 g/100 g in another study [26]. The small difference between them may be related to the variety of carob or the process conditions during flour production. According to a study in the literature, the protein content of raw hazelnut was found as 15.35 g/100 g; therefore, it was concluded that hazelnut flour can be considered a considerable protein source [18]. Our results were also close to the literature findings [27,28]. The hazelnut flour used in this study has testa and it is not defatted. The protein content of flours can vary due to factors such as the variety of the plant, the geographical area where the plant grows, the plant’s growth period, and potentially, the process conditions. The hazelnut flour has a fat content (69.34 g/100 g) that is almost seven times higher than the chickpea flour. The oil content of raw hazelnuts was reported as in the range of 57.65–69.4% in the literature [18]. The lowest fat content (1.47 g/100 g) among the three flours used in this study belongs to carob flour and the result is consistent with a finding in the literature [29]. The hazelnut flour has the highest crude fiber content (22.59 g/100 g) among the others. It was reported that the testa of the hazelnut contains fiber as well as certain phenolic compounds with antioxidant properties [30]. According to the literature, fiber has an influence on the rheological attributes and quality characteristics of the baked goods [31]. It was concluded that legumes with darker seeds have more total phenolic contents than those with lighter colors which is supportive of our results since the carob flour has a very high phenolic composition (24.14 mg/g flour) compared to chickpea flour (0.46 mg/g). It was stated that the antioxidant capacity of legumes has a strong connection with their total phenolic content [32].

The physical characteristics of the flours used in the cookie formulations are also listed in Table 2. The bulk density has a variation between 0.48 to 0.78 g/mL. The hazelnut flour has the lowest bulk density while the carob flour has the highest value. The bulk density of seed flours is primarily influenced by two variables: particle size and packing density. When lipids are present, particles may pack tighter because the triglycerides may function as adhesives in the agglomeration of protein and carbohydrate molecules (either individually or jointly), allowing for higher bulk densities [33]. Although hazelnut flour has high fat content its bulk density is not that high, and this may be related to the relatively large particle size of this flour. The water retention capacity is an important technological attribute in bakery applications. The existence of various hydrophilic carbohydrates and various protein structures could lead to a different water retention capacity of the flours [34]. The pre-cooked chickpea flour has the highest value with 300.76% compared to other two flours. The water retention capacity of the hazelnut flour could not be quantified most likely because of the high fat content of this flour. The carob flour has about 131.85% water retention capacity. According to the literature, because of the hydrophilicity and strong gelling abilities of the carob soluble fiber, carob flour blends have shown very impressive technical capabilities. In comparison to wheat flour, these blends in particular have more water absorption [35]. As far as the oil absorption capacity is concerned, the hazelnut flour has the highest value followed by the chickpea flour. For flavor retention and improved palatability, the oil holding capacity is a critical parameter. It was reported that the existence of non-polar chains, which can interact with lipid hydrocarbon chains to produce hydrophobic interactions, may be responsible for the variation in the oil binding ability of the flours and the primary factor in oil absorption is identified as hydrophobic proteins [5]. The oil absorption capacity of defatted raw hazelnut flour was determined as 1.11 g/g flour which was lower than our result (1.69 g/g flour) [18] and the difference may be related to the defatting process used in the referenced study. This finding points to the potential of hazelnut flour as a taste-stabilizing agent, a property which can be attributed to a system with a high oil absorption capacity. According to the statistical analysis, the emulsion-forming activity and stability values of all the tested flours are close to each other. The emulsion activity values vary between 50.53 and 54.01% and the emulsion stability values change between 91.73 and 98.53%. As far as the stability of the emulsions are concerned, the carob and hazelnut flours have similar foaming capacity (12–12.15%) while the chickpea flour has a higher stability (17.29%) compared to them. Foaming stability did not have much variation with respect to the flour type. The color of the flour is a critical attribute that determines the color of the final product containing this flour, and the flour color depends on the color of the raw material, particle size, and ash concentration. The carob flour has the lowest L* value due to its dark brown color and it has also the highest a* value. Moreover, the hazelnut flour with testa particles has the highest b* value which is very close to the b* value of the carob flour.

### 3.2. Rheological Characteristics of Cookie Doughs

Each type of flour (pre-cooked chickpea, carob, hazelnut) has its own taste, smell, and aroma as well as distinct technological properties. In a previous study, the techno-functional and in vitro digestion properties of a cookie formulation containing only pre-cooked chickpea flour in comparison to other types of chickpea flours (raw and germinated) were determined [19]. In this study, it was thought that the use of three flours together would contribute to the general taste, textural, and rheological properties of the cookies and the mixture design approach was used to investigate the triple flour formulations.

One of the properties that was determined for the cookie formulations (Table 1) is the rheological properties of the doughs (Table 3) and the back extrusion method was used for this purpose. Back extrusion involves the compression of a sample within a cylindrical cell using a plunger that has a loose fit, allowing the sample to pass through the space between the plunger and the wall. The viscoelastic characteristics of the material can be evaluated with this measurement method [36].

Formulation 6 has the lowest firmness (1.23 N), consistency (4.43 N·s), cohesiveness (0.90 N), and viscosity index (0.95 N·s) values. Since the hazelnut flour content (60%) of this dough is the highest among all formulations, low rheological properties are expected. Hazelnut flour has a very high fat content, and this provides a softer texture to the cookie dough. Formulation 1 which consists of 60% pre-cooked chickpea, 15% hazelnut flour, and 15% carob flour has the highest firmness (7.37 N), consistency (24.55 N·s), and cohesiveness (4.19 N) values. Formulation 4 with 15% chickpea, 60% carob, and 15% hazelnut flours has also rheological properties with relatively high values. All of the measured rheological parameters of the triple flour cookies are much lower compared to the cookie made using only pre-cooked chickpea flour [19]. For instance, the reported firmness of the cookie dough having only pre-cooked chickpea flour (40.23 N) is almost 33 times higher than the cookie dough with the highest percentage of hazelnut flour in this study (formulation 6).

**Figure 1:**
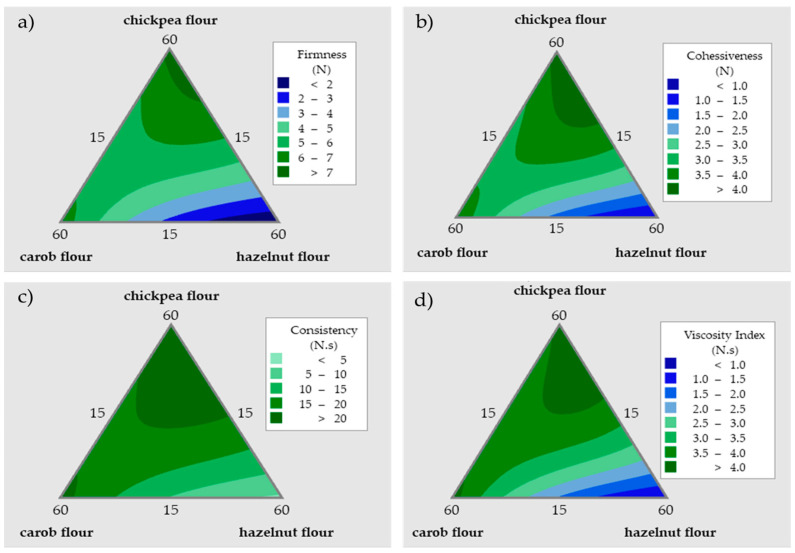
Contour plots showing the effects of flour ratios on the (**a**) firmness, (**b**) cohesiveness, (**c**) consistency, and (**d**) viscosity index of the cookie doughs.

A mixture design analysis was applied to the results of the rheological properties which are firmness, cohesiveness, consistency, and viscosity index. The statistical models and the statistical parameters of the mixture design analysis for the rheological measurements are presented as Supplementary Material (Table S1). The developed models have high R^2^_adjusted_ values for firmness (95.6%), cohesiveness (93.2%), consistency (92.5%), and viscosity index (92.8%) and the models are significant with *p* < 0.05. All parameters have very similar trends as far as the effect of the variables are concerned and contour plots showing the changes in the rheological parameters with respect to the levels of the three flour combinations are given in Figure 1. The change in the firmness of the cookie doughs with varying levels of the flours is shown in Figure 1a. In this plot, the dark blue color refers to the values lower than 2 N and the dark green refers to the values greater than 7 N. As indicated by the contour plot, the higher levels of hazelnut flour can be associated with the reduced firmness of the dough. In addition, elevating the proportion of the pre-cooked chickpea flour resulted in an increase in the firmness levels. The contour plot shown in Figure 1b indicates the change in the cohesiveness values of the cookie doughs. In this plot, the darkest blue part represents values lower than 1 N, and the tone of green becomes darker while the cohesiveness value is increased. As can be seen from the graph, when the amount of the hazelnut flour is increased up to 60%, the cohesiveness value becomes lower. The increased amounts of the carob and chickpea flours make the cookie dough more cohesive. For the contour plot belonging to consistency, the lightest green refers to the values lower than 5 N, and the green color becomes darker and darker while the consistency value is increasing (Figure 1c). The high amounts of carob and chickpea flour addition (up to 60%) cause the cookie dough samples to become more consistent. On the other hand, the high amount of hazelnut flour (up to 60%) results in lower consistency values. According to the contour plot of the viscosity index, the dark blue color indicates the viscosity index which is lower than 1 N, and the darkest green is the viscosity index values that are higher than 4 N (Figure 1d). The same trend also applies to this parameter as it is valid for the other three rheological properties. The increase in the carob and chickpea flour results in an increase in the viscosity index while the opposite is true for the hazelnut flour.

As in the current study, it was reported that hazelnut flour addition to wheat flour in bread formulations resulted in lower rheological parameters due to a weakening effect on the dough structure [37]. On the other hand, including carob flour in a bread recipe containing rice flour caused an increase in the elastic attribute of the product and the strength of the bread because of the fiber content of carob flour [8]. This result is also in parallel with our findings. Giving careful consideration to the rheology of the dough is essential during the process of formulating the cookies. Very soft or very firm cookie doughs are difficult to work with; therefore, the cookie doughs should possess the right level of cohesion to maintain its structure during the processing, facilitating simple lamination without excessive stickiness that could cause adherence to the rolling mill [38]. By considering all four contour plots and the personal experience during cookie processing, the use of hazelnut flour between 15 and 45% is more appropriate for improving the rheological characteristics of the investigated doughs. The high amounts of hazelnut flour make the dough handling more difficult. Due to its high fat content, it produces a sticky dough which is difficult to shape. Pre-cooked chickpea flour between 30 and 60% provides more ideal results for the rheological attributes of the doughs since it is easier to handle these doughs at these levels. Carob flour, with its high sugar content, makes the cookie dough firmer and non-sticky which makes it easier to handle and shape. As the results indicate, pre-cooked chickpea flour addition also increased the rheological parameters of the cookie doughs, especially the firmness and consistency values.

### 3.3. Technological Attributes of the Baked Cookies

The moisture, baking weight loss, spread factor, hardness, and color properties are determined as the technological properties for the baked cookies (Table 4). A simplex-centroid mixture design shown in Table 1 was applied and the results were analyzed statistically. The results of the statistical models and the model parameters are provided in Table S2 (Supplementary Material). The constructed models for the moisture, the baking weight loss, the spread factor, and the hardness have R^2^_adjusted_ values of 58.8%, 62.7%, 95.5%, and 70.1%. All the models are significant with *p* < 0.05. However, the models for the baking weight loss and the hardness have significant lack of fit and this situation could arise due to either the model’s predictive performance, the low variation in replications, or a combination of both reasons.

**Figure 2:**
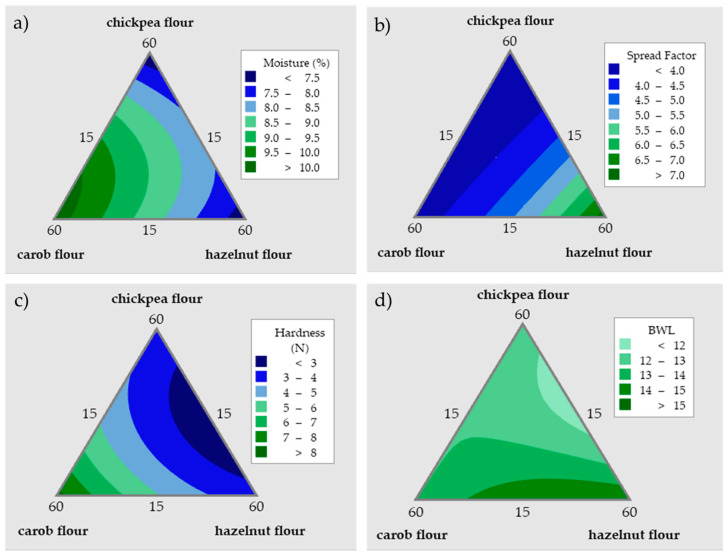
Contour plots showing the effects of the flour ratios on the (**a**) moisture content, (**b**) spread factor, (**c**) hardness, and (**d**) baking weight loss (BWL) of the baked cookies.

The texture of the cookies and their customer acceptance are significantly influenced by the moisture level of cookie-type products. According to Table 4, formulation 4 has the highest and formulation 1 has the lowest moisture contents. However, there was not much difference between the moisture levels of the products formed with the triple flour formulations. The moisture levels of the cookies ranged between 7.16 and 10.28%. Figure 2 represents the contour plots of the results of the mixture design and for the contour plot of moisture (Figure 2a), the dark blue color shows the percent moisture content lower than 7.5% and dark green indicates the moisture content higher than 10%. According to this plot, the higher amounts of hazelnut and chickpea flours caused lower moisture content for the cookies. The high amount of carob flour, on the other hand, resulted in a higher moisture content. Chickpea flour, with its high protein and starch contents, could lead to an interaction between these compounds, and water can associate tightly with these structures, while the high oil content of hazelnut flour can cause a repelling effect and evaporation of water. Carob flour, on the other hand, is richer in terms of mono- and oligosaccharides which have the capability to interact with water but not through 3D networks.

One of the factors which helps to estimate the cookie quality attributes is the spread factor. A higher spread factor is ideal for better cookies [39]. The dough viscosity appears to be an important factor in the cookie spread rate. Therefore, a correlation between the viscosity index and spread factor values were investigated and the R^2^ value was determined as 0.88 which indicates a good relation between these two parameters. An increased water content in the dough results in greater sugar dissolution during mixing. A lower initial dough viscosity facilitates faster cookie spreading during the baking process. Flour components with a high water absorption capacity lower the water level which is available for sugar dissolution in the recipe. As a result, the dough has a higher initial viscosity and spreads less while baking. Cookies with a greater spread are made with flours with poor hydration qualities [40]. In a study, it was concluded that the viscosity of the dough affected how quickly the cookies spread during baking [41]. In the current study, the spread factor values ranged between 3.37 and 7.47. Only formulation 6, which is a cookie consisting of 60% hazelnut flour together with 15% chickpea and 15% carob flours, has a spread factor with a large difference in comparison with the other samples. This formulation has a high hazelnut flour content with a poor water retention capacity; therefore, the high spread factor of this formulation can be associated with this characteristic of hazelnut flour. Formulation 4, consisting of 60% carob flour, has the lowest spread factor. Other cookie samples have similar spread factor results. According to the contour plot in Figure 2b which represents the result of the mixture design for the spread factor, the dark blue color indicates the values lower than four and the darkest green color indicates a value greater than seven. An almost linear relationship can be observed between the amount of hazelnut flour and the spread factor. While the amount of hazelnut flour increased, the spread factor of the cookie also increased. The opposite is true for the relationship between the carob flour and the spread factor. There is an inverse relation between the amount of carob flour and the spread factor, and as the amount of carob flour increases, the spread factor of the cookie decreases. This result is contrary to what was observed in a study involving an investigation for biscuits containing carob flour and dry apple pomace [42]. Interactions among the ingredients may cause this difference in the effect of carob flour on spread rate.

The hardness values as the textural attribute of the cookies made with the triple flour mixtures were determined and are shown in Table 4. The hardness values of the cookies have a range of 2.56 N to 8.26 N. Formulation 4 which consists of 60% carob flour has the highest hardness value (8.26 N) and formulation 5 which had 37.5% of carob flour has the second highest hardness value (5.84 N). Formulation 6 had a high amount of hazelnut flour; hence, its fat content was also high. This situation caused the cookie to have a texture that can hardly be divided into two pieces. According to the contour plot of hardness (Figure 2c), the dark blue color indicates a hardness value smaller than 3 N and the darkest green represents a hardness value greater than 8 N. It is observed that as the amount of carob flour increases up to 60%, the hardness value also increases and even reaches its highest values. This result is comparable with the findings obtained in another study that investigated a cookie formulation containing carob flour [42]. If the amounts of chickpea flour and hazelnut flour are increased, this causes a lowering effect in the hardness value of the cookie. Compared to the triple flour cookies investigated in this study, the hardness value (4.5 N) of the cookie made only from pre-cooked chickpea flour was higher, except formulation 4 and 5, both of which have 15% chickpea flour [19].

The BWL is an important quality attribute for bakery products to understand how much loss takes place from the dough to the baked product. During baking, cookies lose moisture; therefore, there is a change in the structure and texture, and an increase in the volume. The primary mechanisms in the formation of crumbs are generally recognized to be moisture losses and starch retrogradation. A dry crust may form if too much moisture is removed, which will make the product lighter, causing a more difficult packaging application. Additionally, the moisture lost during baking has a negative consequence on the freshness of the baked items, causing them to age more quickly [43]. In this study, the baking weight loss values had a range between 11.73 and 19.93%. The highest value was observed for sample 6 which was a cookie consisting of 60% hazelnut flour. The results of the other samples were close to each other. According to the mixture contour plot of BWL in Figure 2d, the lightest green indicates that a BWL lower than 12% and the darkest green shows the values greater than 15%. If the amount of hazelnut flour was increased, % loss also increased. In addition, a high amount of carob flour usage also causes higher BWL values.

The color is one of the major quality characteristics for the cookies that influences whether a consumer will accept the finished product. According to Table 4, formulation 1 has the highest L* value (41.42) which is an expected result since this formulation has the highest pre-cooked chickpea flour content (60%) and chickpea flour has the lightest color compared to hazelnut and carob flours. Conversely, formulation 4 has the lowest L* value (25.68) since it has the highest carob flour content. Redness is shown with a* values and yellowness with b* values in the color parameter determination of the samples. The a* values of the cookies were found to be similar. However, formulations 7, 8, and 9, which are the central points in the mixture design, have the highest a* values (9.98, 9.92, 9.76). Formulation 1, which consists of the highest amount of chickpea flour, has the highest b* value (13.77) and formulation 4, which has the highest amount of carob flour, has the lowest b* value (6.73). Besides the color of the raw material, the reactions that take place while baking also make the cookies darker.

### 3.4. Sensory Properties of Baked Cookies

Since the purpose of combining three different flours in the cookie formulation is to improve the taste and the flavor issues of the cookie containing only chickpea flour, the cookies containing three flours were compared with chickpea flour cookies in the sensory analysis. The chickpea flour cookie has the same formulation as the triple flour formulations except that it contains only pre-cooked chickpea flour [19]. The two other cookies selected to be tasted in the sensory analysis were chosen based on the test results and the personal experience during their preparation. Parameters such as the handling characteristics of the dough and the textural attributes of the cookie played an important role in this selection. As a result, cookies with 30% chickpea flour, 30% carob flour, and 30% hazelnut flour (central point samples 7, 8 and 9), and those with 60% chickpea flour, 15% carob flour, and 15% hazelnut flour (sample 1) were tested in the sensory analysis.

The results of the sensory test are provided in Table 5. There was no statistical difference in the color and texture attributes of all three cookies. Formulation 7 has the highest scores for flavor, taste and acceptability. The sample which contains only the pre-cooked chickpea flour has the lowest flavor score. This is an expected result considering the undesirable flavor of chickpea flour by consumers as also indicated in another study [15]. This cookie also has the lowest scores for taste and the overall acceptability parameters. Formulation 1 has similar taste and flavor scores to formulation 7. From the consumer point of view, sample 7 has the highest overall acceptability. In particular, the sensory attributes of the cookies containing carob and hazelnut flours were improved. The carob flour introduced a nice cocoa-like flavor to the product, while the hazelnut flour contributed a pleasing and compatible flavor profile, exceptionally suitable for bakery items. Therefore, the conclusion can be reached that including carob and hazelnut flours to chickpea flour-containing formulations increases the appreciation and preference of the consumer compared to the cookies containing only pre-cooked chickpea flour.

### 3.5. Nutritional Characteristics of the Baked Cookies

The gluten-free cookie that was identified as having the best overall acceptability (formulation with 30% chickpea, 30% hazelnut, and 30% carob flour) as a result of the sensory evaluation was also investigated regarding its predicted nutritional value. For this purpose, Atwater general factors were used to assess the food’s energy content. This method was used to calculate the total energy of 100 g of the cookie in kcal. For this calculation, the amount of carbohydrate, protein, fat, and fiber are considered as 4 kcals/g, 4 kcals/g, 9 kcals/g, and 2 kcals/g, respectively. This particular cookie provides 510.9 kcal/100 g and 19.9% of this energy comes from the protein. It was reported that the cookie made from only pre-cooked chickpea flour provides 476.5 kcal/100 g and 24.7% of this energy comes from the protein [19]. Therefore, the triple flour cookie combination supplies more energy compared to the cookie made with only chickpea flour.

Additionally, the digestible starch fractions of the cookie with 30% chickpea, 30% hazelnut, and 30% carob flour formulation were determined with an in vitro digestion analysis. The rapidly digestible, slowly digestible, and resistant starch fractions of this cookie were determined as 16.82 ± 3.17 g/100 g dry basis, 5.36 ± 3.51 g/100 g dry basis, 8.30 ± 0.1 g/100 g dry basis, respectively. In terms of percentages, these values correspond to 55.2% for rapidly digestible starch, 17.6% for slowly digestible starch, and 27.2% for resistant starch. These ratios are similar to those reported for the cookie with only pre-cooked chickpea flour (54.5% rapidly digestible starch, 16.5% slowly digestible starch, and 29% resistant starch) [19]. Hence, it can be concluded that although adding carob and hazelnut flour to the cookie formulation reduced the amount of starch, it did not cause major changes in the different starch fraction ratios.

## 4. Conclusions

The addition of carob and hazelnut flours to cooked chickpea flour in a cookie formulation was investigated with a mixture design. The compositional differences directly influence both the sensory and the physical characteristics of the cookie samples to which they are added. Hazelnut flour has a very high fiber content, which is one of the most critical parameters having an influence on the consumer’s preference. Carob flour differs from the other flours with its high phenolic content and added desirable color, flavor, and taste characteristics. The rheological properties of the cookie doughs are reduced when the hazelnut and the carob flour are added. A direct relation between the increasing amounts of the hazelnut flour and spread factor was found while the carob flour caused a decrease in the same parameter. The cookies made with three flours displayed reduced hardness as the amount of the hazelnut flour increased. In sum, addition of the carob and the hazelnut flours improved the overall acceptability of the pre-cooked chickpea flour cookie.

In light of these findings, there is a potential for exploring novel applications of the chickpea, carob, and hazelnut flours employed in this study. In addition, thanks to their high nutritional characteristics, gluten-free products produced with these flours can serve as an alternative for the low-nutrition gluten-free products on the market. Every type of flour possesses its unique advantages and limitations, making them suitable for specific proportions in formulations. An investigation of the incorporation of chickpea flour with various nut flours and/or non-legume flours could be a valuable avenue to diversify the range of gluten-free bakery products. Furthermore, the flours examined in this study could find utility in various other bakery applications.

## Figures and Tables

**Table 1:** Ingredients of the cookies formulated with simplex-centroid mixture design *.

Ingredients (g)	Formulations								
	1	2	3	4	5	6	7	8	9
Chickpea flour	60	37.5	37.5	15	15	15	30	30	30
Carob flour	15	37.5	15	60	37.5	15	30	30	30
Hazelnut flour	15	15	37.5	15	37.5	60	30	30	30
Corn starch	10	10	10	10	10	10	10	10	10
Brown sugar	40	40	40	40	40	40	40	40	40
Margarine	60	60	60	60	60	60	60	60	60
Egg	60	60	60	60	60	60	60	60	60
Baking powder	10	10	10	10	10	10	10	10	10

* Sum of all flours and corn starch is 100 g for all formulations.

**Table 2:** Chemical and physical characteristics of the flours used in the cookie formulations.

Properties	Pre-Cooked Chickpea	Carob	Hazelnut with Testa
Moisture (g/100 g)	3.30 ± 0.20 ^a^	1.82 ± 0.26 ^b^	1.06 ± 0.18 ^b^
Protein (g/100 g)	20.71 ± 1.60 ^a^	3.66 ± 0.75 ^c^	15.65 ± 0.09 ^b^
Fat (g/100 g)	10.06 ± 1.27 ^b^	1.47 ± 1.11 ^c^	69.34 ± 0.86 ^a^
Ash (g/100 g)	1.95 ± 0.11 ^b^	3.36± 1.12 ^a^	1.98 ± 0.04 ^b^
Fiber (g/100 g)	1.96 ± 0.07 ^b^	1.09 ± 0.34 ^c^	22.59 ± 0.22 ^a^
Total Phenolic Content (mg GAE/g flour)	0.46 ± 0.08 ^c^	24.14 ± 5.72 ^a^	1.83 ± 0.00 ^b^
Bulk Density (g/mL)	0.70 ± 0.00 ^a^	0.78 ± 0.04 ^a^	0.48 ± 0.00 ^b^
Water Retention Capacity (%)	300.76 ± 12.84 ^a^	131.85 ± 6.30 ^b^	-
Oil Absorption Capacity (g/g)	1.38 ± 0.13 ^ab^	1.13 ± 0.10 ^b^	1.69 ± 0.21 ^a^
Emulsion Activity (%)	51.00 ± 1.41 ^a^	50.53 ± 3.68 ^a^	54.01 ± 4.23 ^a^
Emulsion Stability (%)	95.15 ± 4.03 ^a^	98.53 ± 2.08 ^a^	91.73 ± 6.03 ^a^
Foaming Capacity (%)	17.29 ± 3.83 ^a^	12.15 ± 5.15 ^b^	12.00 ± 0.00 ^b^
Foaming Stability (%)	8.17 ± 0.24 ^a^	6.87 ± 0.70 ^a^	2.83 ± 2.00 ^a^
L*	90.60 ± 1.01 ^a^	58.68 ± 1.38 ^c^	75.89 ± 0.58 ^b^
a*	0.91 ± 0.06 ^c^	12.29 ± 0.12 ^a^	5.92 ± 0.05 ^b^
b*	31.77 ± 0.23 ^a^	29.26 ± 0.56 ^b^	30.27 ± 0.13 ^ab^

Values are mean ± SD. Significant differences in means are indicated by distinct letters within the same row (*p* < 0.05).

**Table 3:** Rheological properties of cookie doughs containing pre-cooked chickpea, carob, and hazelnut flours.

Formulation	Firmness (N)	Consistency (N × s)	Cohesiveness (N)	Viscosity Index (N × s)
1	7.37 ± 0.48 ^a^	24.55 ± 1.37 ^a^	4.19 ± 0.23 ^a^	4.13 ± 0.06 ^a^
2	5.44 ± 0.23 ^c^	18.46 ± 0.26 ^b^	3.34 ± 0.04 ^b^	3.66 ± 0.17 ^a^
3	5.17 ± 1.40 ^b,c^	19.32 ± 2.11 ^a,b^	3.29 ± 1.05 ^a,b^	3.10 ± 1.28 ^a^
4	6.62 ± 0.74 ^a,b^	21.91 ± 3.7 ^a,b^	3.92 ± 0.47 ^a,b^	4.22 ± 0.47 ^a^
5	2.66 ± 0.14 ^d^	9.33 ± 0.82 ^c^	1.85 ± 0.12 ^b^	1.93 ± 0.13 ^b^
6	1.23 ± 0.03 ^e^	4.43 ± 0.09 ^c^	0.9 ± 0.0 ^d^	0.95 ± 0.04 ^c^
7	5.88 ± 0.19 ^b,c^	20.03 ± 1.22 ^a,b^	3.80 ± 0.19 ^a,b^	3.93 ± 0.21 ^a^
8	5.39 ± 0.31 ^c^	19.31 ± 1.39 ^b^	3.30 ± 0.25 ^b^	3.45 ± 0.31 ^a^
9	5.31 ± 0.24 ^c^	18.16 ± 1.61 ^b^	3.39 ± 0.22 ^b^	3.67 ± 0.3 ^a^

Values are mean ± standard deviation. Significant differences in means are indicated by distinct letters within the same column (*p* < 0.05).

**Table 4:** Characteristics of the cookies containing combinations of pre-cooked chickpea, carob, and hazelnut flours.

Formulation	Moisture (%)	Baking Weight Loss (%)	Spread Factor	Hardness	L*	a*	b*
1	7.16 ± 0.56 ^e^	13.05 ± 0.58 ^b,c^	3.77 ± 0.15 ^f^	3.92 ± 0.55 ^c,d^	41.42 ± 1.32 ^a^	9.06 ± 0.80	13.77 ± 0.67 ^a^
2	9.13 ± 0.23 ^a,b,c^	12.87 ± 0.80 ^b^	3.87 ± 0.17 ^f^	4.82 ± 0.58 ^b,c^	30.16 ± 1.74 ^c,d^	9.70 ± 0.40 ^a^	9.72 ± 0.56 ^a,b,c^
3	7.77 ± 0.30 ^d,e^	12.85 ± 1.13 ^d^	4.43 ± 0.30 ^c^	2.86 ± 0.53 ^d,e^	39.43 ± 1.72 ^a,b^	9.15± 0.72 ^a^	12.91 ± 1.01 ^a,b^
4	10.28 ± 0.13 ^a^	13.59 ± 0.75 ^b,c^	3.37 ± 0.14 ^e,f^	8.26 ± 1.21 ^a^	25.68 ± 1.51 ^c,d^	8.95 ± 0.35 ^a^	6.73 ± 0.74 ^c^
5	8.49 ± 0.25 ^c,d,e^	16.46 ± 0.87 ^a^	4.92 ± 0.32 ^b^	5.84 ± 0.82 ^b^	28.18 ± 0.76 ^c,d^	9.55 ± 0.24 ^a^	8.28 ± 0.86 ^b,c^
6	7.24 ± 0.17 ^e^	19.93 ± 1.10 ^a^	7.47 ± 0.38 ^a^	3.45 ± 0.89 ^d,e^	34.27 ± 1.53 ^a,b,c^	8.88 ± 0.57 ^a^	11.30 ± 1.27 ^a,b,c^
7	8.94 ± 0.19 ^b,c,d^	12.60 ± 0.92 ^d^	4.05 ± 0.20 ^d,e^	3.75 ± 0.70 ^d,e^	32.68 ± 1.23 ^b,c,d^	9.98 ± 0.31 ^a^	10.45 ± 0.51 ^a,b,c^
8	8.69 ± 0.35 ^c,d^	13.87 ± 1.10 ^b^	4.27 ± 0.15 ^c,d^	3.61 ± 0.63 ^d,e^	31.15 ± 1.50 ^c,d^	9.92 ± 0.43 ^a^	10.11 ± 0.65 ^a,b,c^
9	10.04 ± 0.37 ^a,b^	11.73 ± 0.65 ^c,d^	4.15 ± 0.20 ^d,e^	2.56 ± 0.26 ^e^	31.03 ± 1.09 ^c,d^	9.76 ± 0.62 ^a^	10.33 ± 0.98 ^a,b,c^

Values are mean ± standard deviation. Significant differences in means are indicated by distinct letters within the same column (*p* < 0.05).

**Table 5:** Sensory test scores of the cookies *.

Samples	Chickpea Flour	30% Chickpea/30% Carob/30% Hazelnut Flours (Formulation 7)	60% Chickpea/15% Carob/15% Hazelnut Flours (Formulation 1)
Color	5.60 ± 1.15 ^a^	5.80 ± 1.36 ^a^	5.43 ± 1.28 ^a^
Flavor	4.50 ± 1.43 ^b^	5.40 ± 1.33 ^a^	5.33 ± 1.25 ^a^
Texture	5.48 ± 1.06 ^a^	5.43 ± 1.44 ^a^	5.50 ± 0.98 ^a^
Taste	4.55 ± 1.41 ^b^	5.55 ± 1.17 ^a^	5.43 ± 1.03 ^a^
Overall acceptability	4.78 ± 1.19 ^b^	5.63 ± 1.05 ^a^	5.28 ± 1.11 ^ab^

* 1–7 scale. Values are mean ± SD. Significant differences in means are indicated by distinct letters within the same row (*p* < 0.05).

## Data Availability

The data used to support the findings of this study can be made available by the corresponding author upon request.

## Supplementary Materials

The following supporting information can be downloaded at: https://www.mdpi.com/article/10.3390/foods12193689/s1, Table S1: Statistical parameters of simplex-centroid mixture design models for the rheological properties of the cookie doughs obtained from analysis of variance; Table S2: Statistical parameters of simplex-centroid mixture design models for the technological properties of the baked cookies obtained from analysis of variance.
