# Supplementary material for: Formulation of Gluten-Free Cookies Utilizing Chickpea, Carob, and Hazelnut Flours through Mixture Design
Source: Foods. 2023 Oct 8;12(19):3689. doi: 10.3390/foods12193689 (PMC10572517; doi:10.3390/foods12193689)
Supplement: Supplementary file 1 [file foods-12-03689-s001.zip › foods-2640487-supplementary.pdf]

Supplementary material: Dogruer et al., Formulation of Gluten-Free Cookies Utilizing Chickpea, Carob, and Hazelnut Flours Through Mixture Design

Table S1. Statistical parameters of simplex-centroid mixture design models for the rheological properties of the cookie doughs obtained from analysis of variance*

Response	Model**	R^2	R^2adj	p model
Consistency	$0.533*x_1+0.743*x_2+0.058*x_3-0.026*x_1*x_2-0.006*x_1*x_3-0.024x_2*x_3+0.001*x_1*x_2*x_3$	94.3	92.5	0.000
Firmness	$0.153*x_1+0.222*x_2+0.008*x_3-0.007*x_1*x_2-0.001*x_1*x_3-0.007*x_2*x_3$	96.7	95.6	0.000
Viscosity index	$0.066*x_1+0.124*x_2-0.001x_3-0.003*x_1*x_2-0.004*x_1*x_3$	94.5	92.8	0.000
Cohesiveness	$0.067*x_1+0.117*x_2-0.01*x_3-0.003*x_1*x_2+0.001*x_1*x_3-0.003*x_2*x_3$	94.8	93.2	0.000

* It's not possible to calculate the lack-of-fit test for this particular design because mixture model has already utilized all the available degrees of freedom.

* x_1: chickpea flour; x_2: carob flour; x_3: hazelnut flour

Table S2. Statistical parameters of simplex-centroid mixture design models for the technological properties of the baked cookies obtained from analysis of variance

Response	Model*	R^2	R^2adj	p model	p lack of fit
Moisture	$0.022*x_1+0.122*x_2+0.049*x_3+0.002*x_1*x_2+0.002*x_1*x_3$	61.9	58.8	0.000	0.1
Baking weight loss	$0.198*x_1+0.105*x_2+0.218*x_3-0.005*x_1*x_3+0.002*x_2*x_3$	64.2	62.7	0.000	0.000
Spread ratio	$0.048*x_1+0.039*x_2+0.147x_3-0.002*x_1*x_3-0.001*x_2*x_3$	95.7	95.5	0.000	0.467
Hardness	$0.1*x_1+0.178*x_2+0.064*x_3-0.003*x_1*x_2-0.003*x_1*x_3-0.002*x_2*x_3$	71.6	70.1	0.000	0.000

* x_1: chickpea flour; x_2: carob flour; x_3: hazelnut flour
